# Associations between *MTHFR* gene polymorphisms and the risk of intracranial hemorrhage: Evidence from a meta‐analysis

**DOI:** 10.1002/brb3.1840

**Published:** 2020-11-27

**Authors:** Fenghui Wang, Zhendong Xu, Haiyan Jiao, Aixiang Wang, Youbin Jing

**Affiliations:** ^1^ Department of Neurosurgery Pingdu People's Hospital Affiliated to Weifang Medical College Pingdu China; ^2^ Department of Laboratory Medicine Qingdao Blood Center Qingdao China; ^3^ Department of Laboratory Medicine Pingdu People's Hospital Affiliated to Weifang Medical College Pingdu China

**Keywords:** cerebral hemorrhage, gene polymorphisms, intracranial hemorrhage, intraventricular hemorrhage, meta‐analysis, methylenetetrahydrofolate reductase

## Abstract

**Introduction:**

Previously, a number of genetic epidemiological studies have evaluated associations between *MTHFR* gene polymorphisms and the risk of intracranial hemorrhage (ICH), with controversial results. Accordingly, we carried out this meta‐analysis to more conclusively evaluate associations between *MTHFR* gene polymorphisms and the risk of ICH.

**Methods:**

MEDLINE, EMBASE, Wanfang, VIP, and CNKI were searched comprehensively, and thirty‐one genetic association studies were finally selected to be included in this meta‐analysis.

**Results:**

Eight literatures (963 cases and 2,244 controls) assessed relationship between *MTHFR* rs1801131 (A1298C) polymorphism and the risk of ICH, and thirty‐one literatures (3,679 cases and 9,067 controls) assessed relationship between *MTHFR* rs1801133 (C677T) polymorphism and the risk of ICH. We found that AA genotype of rs1801131 polymorphism was significantly associated with a decreased risk of intraventricular hemorrhage (IH) compared with AC/CC genotypes (OR = 0.63; *p* = .003), AC genotype was significantly associated with an increased risk of IH compared with AA/CC genotypes (OR = 1.55; *p* = .005), and A allele was significantly associated with a decreased risk of IH compared with C allele (OR = 0.75; *p* = .02). Additionally, CC genotype of rs1801133 polymorphism was significantly associated with a decreased risk of cerebral hemorrhage (CH) compared with CT/TT genotypes (OR = 0.75; *p* = .04), TT genotype was significantly associated with an increased risk of CH compared with CC/CT genotypes (OR = 1.27; *p* = .02), and C allele was significantly associated with a decreased risk of CH compared with T allele (OR = 0.85; *p* = .007).

**Conclusions:**

This meta‐analysis shows that rs1801131 polymorphism may influence the risk of IH, while rs1801133 polymorphism may influence the risk of CH.

## Graphical abstract

This is a comprehensive meta‐analysis about polymorphisms in MTHFR gene and ICH. We found that rs1801131 (A1298C) polymorphism was significantly associated with the risk of IH. We found that rs1801133 (C677T) polymorphism was significantly associated with the risk of CH.

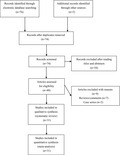

## 1 INTRODUCTION

Intracranial hemorrhage (ICH), an acute cerebral vascular disorder characterized by nontraumatic bleeding into brain parenchyma, has high death and disability rates (Collaborators, 2015; Global Burden of Disease Study, 2013). ICH can be divided into subarachnoid hemorrhage (SAH), intraventricular hemorrhage (IH), cerebral hemorrhage (CH), and hemorrhagic stroke (HS). SAH refers to spontaneous bleeding into subarachnoid space, IH refers to spontaneous bleeding into ventricular system, CH refers to spontaneous bleeding into brain parenchyma, and HS refers to loss of blood supply to brain parenchyma caused by rupture of blood vessels (may result in spontaneous bleeding into subarachnoid space, subdural space, or brain parenchyma). Although the incidence of ICH is found to be relatively lower than ischemic cerebral vascular disorder, the associated mortality and disability rates of ICH are actually much higher than that of ischemic cerebral vascular disorder (Caceres & Goldstein, 2012; Freeman & Aguilar, 2012). So far, the disease mechanisms of ICH are still not clear. Nevertheless, the remarkable differences observed in incidences of ICH among different ethnic groups by previous epidemiological investigations indicated that genetic architecture may play a vital role in its development (Backhaus et al., 2015; Krishnan et al., 2018).

Methylenetetrahydrofolate reductase (MTHFR) regulates the metabolism of folate and homocysteine (Moll & Varga, 2015; Zaric et al., 2019). Previous experimental studies have demonstrated that deficiency of MTHFR could lead to hyperhomocysteinemia and it also give rise to an elevated risk of multiple cardiovascular and cerebrovascular disorders (Luo et al., 2018; Santilli, Davì, & Patrono, 2016; Wei et al., 2017). Accordingly, it is rational to speculate that functional polymorphisms of *MTHFR* gene, which may lead to disruption of homocysteine metabolism and give rise to hyperhomocysteinemia, may also influence the risk of a wide variety of cardiovascular and cerebrovascular disorders. Past experimental studies have found that the C/T variation of rs1801133 and A/C variation of rs1801131 are both associated with alteration of amino acid sequence and reduction in MTHFR enzymatic activity (Li, Dai, Zheng, Liu, & Huang, 2015; Trimmer, 2013). Therefore, it is possible that individuals carrying the mutant allele of *MTHFR* rs1801131 or rs1801133 polymorphism may be more prone to develop hyperhomocysteinemia and its associated vascular disorders.

Previously, many investigators across the world have extensively tried to examine the associations of *MTHFR* rs1801131 and rs1801133 polymorphisms with the risk of ICH, yet with inconsistent results. Accordingly, a meta‐analysis was carried out by us to robustly estimate the associations between those two *MTHFR* polymorphisms and the risk of ICH.

## 2 MATERIALS AND METHODS

This meta‐analysis was conducted in accordance with the PRISMA guideline (Moher, Liberati, & Tetzlaff, 2009).

### 2.1 Literature search and inclusion criteria

MEDLINE, EMBASE, CNKI, Wanfang, and VIP were systematically searched by us using keywords as follows: (Methylenetetrahydrofolate reductase or MTHFR) AND (polymorphic or polymorphism or variant or variation or mutant or mutation or SNP or genotypic or genotype or allelic or allele) AND (Cerebral hemorrhage or subarachnoid hemorrhage or Basal ganglia hemorrhage or Putaminal hemorrhage or Brain hemorrhage or Cerebrum hemorrhage or Intracranial hemorrhage). Moreover, the reference lists of retrieved publications were also screened by us to offset the possible incompleteness of literature searching from electronic databases.

Selection criteria of eligible publications were as follows: (a) case–control or cohort design; (b) give genotypic or allelic frequencies of *MTHFR* polymorphisms in cases with ICH and control subjects; and (c) the full manuscript with genotypic or allelic frequencies of *MTHFR* polymorphisms is freely downloadable or buyable. If duplicate reports by the same group of authors are identified, only the study with the largest sample size would be selected for pooled analyses.

### 2.2 Data extraction and quality assessment

The following data items were extracted from eligible publications: (a) name of the first author; (b) year of publication; (c) country and ethnic background of study subjects; (d) number of cases with ICH and control subjects; and (e) genotypic or allelic frequencies of *MTHFR* polymorphisms in cases with ICH and control subjects. A *p* value of Hardy–Weinberg equilibrium (HWE) was also calculated using genotypic frequencies of *MTHFR* polymorphisms, and the threshold of HWE derivation was set at 0.05. The quality of eligible publications was assessed by the Newcastle–Ottawa scale (NOS) (Stang, 2010), and these with a score of 7–9 were considered to be of good quality. Two authors carried out data extraction and quality assessment in parallel. A thorough discussion until a consensus is reached would be initiated if discrepant results were found between two authors.

### 2.3 Statistical analyses

All statistical analyses in this meta‐analysis were performed with the Cochrane Review Manager software. Associations between MTHFR gene polymorphisms and the risk of ICH were explored by using odds ratio and its 95% confidence interval. The statistically significant *p* value was set at 0.05. All investigated polymorphisms have a major allele (M) and a minor allele (m), the dominant comparison was defined as MM versus Mm + mm, the recessive comparison was defined as mm versus MM + Mm, the overdominant comparison was defined as Mm versus MM + mm, and the allele comparison was defined as M versus m. The authors used *I* ^2^ statistics to examine heterogeneities among included studies. The authors would use DerSimonian–Laird method, which is also known as the random effect model, to pool the results of eligible studies if *I* ^2^ is larger than 50%. Otherwise, the authors would use Mantel–Haenszel method, which is also known as the fixed effect model, to pool the results of eligible studies. Meanwhile, the authors also conduct subgroup analyses by ethnic background and disease subtypes. Sensitivity analyses were conducted by deleting one eligible study each time, and then recalculating the pooled results based on the rest of eligible studies. Publication biases were evaluated with funnel plots and Egger's tests. Meta‐regression analysis was further performed to determine whether study quality of eligible studies can change the effect size.

## 3 RESULTS

### 3.1 Characteristics of included studies

**Figure 1:**
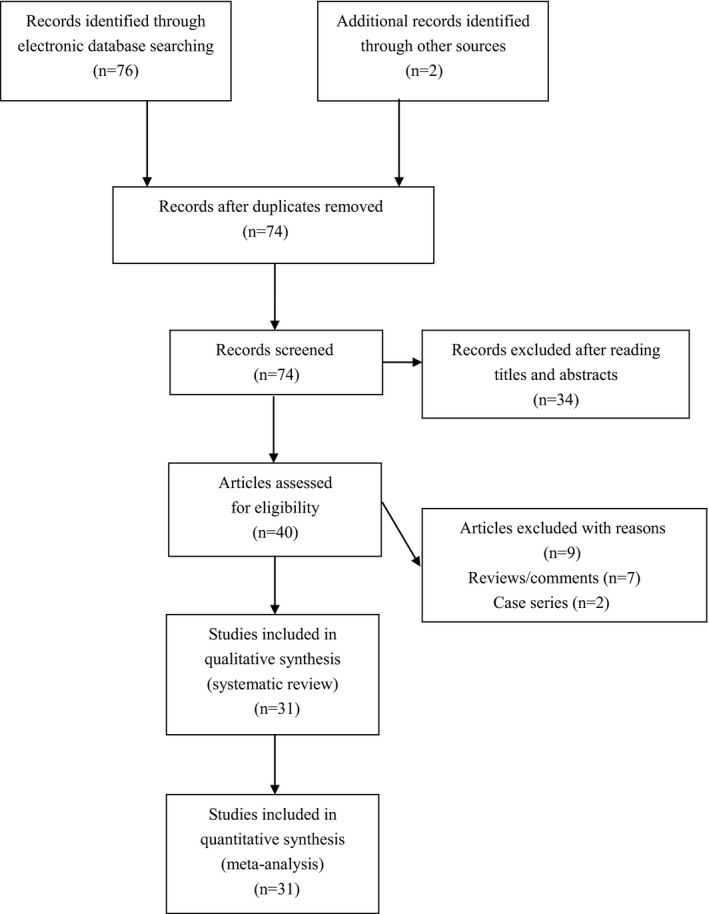
Flowchart of study selection for this meta‐analysis

Seventy‐eight publications were identified by us using our searching strategy. We screened forty publications for eligibility of inclusion after excluding thirty‐four unrelated publications and four repeated reports. Seven reviews and two case series were further excluded. Totally, thirty‐one studies met the selection criteria and were finally enrolled for pooled analyses (Figure 1). Data items extracted from eligible studies were summarized in Table 1. In brief, the eligible studies were published between 1998 and 2018, and they all used healthy blood donors as controls. The NOS score of eligible studies ranged from 7 to 8, which indicated that all eligible studies were of relatively good quality.

**Table 1:** The characteristics of included studies in this meta‐analysis

First author, year	Country	Ethnicity	Type of disease	Sample size Cases/Controls	Sex, male (%) Cases/Controls	Age (years) Cases/Controls	Genotype distribution		*p* value for HWE	NOS score
							Cases	Controls		
rs1801131 A1298C							AA/AC/CC			
Abidi 2018	Morocco	Mixed	HS	113/323	NA	NA	64/46/3	186/120/17	.678	7
Adén 2013	USA	Mixed	IH	224/389	57.1/59.4	NA	98/108/18	213/144/32	.277	7
Dikmen 2006	Turkey	Caucasian	HS	49/55	54.2/29.1	63.4/56.8	19/23/7	19/33/3	.021	7
Hultdin 2011	Sweden	Caucasian	HS	59/767	78.3/58.6	54.8/55.0	29/29/1	328/346/93	.905	8
Kumar 2018	India	Mixed	SAH	100/50	47.0/52.0	50.5/48.0	38/37/25	20/16/14	.013	7
Sazci 2006	Turkey	Caucasian	HS	120/259	55.8/55.6	53.5/56.7	52/46/22	130/108/21	.828	7
Semmler 2008	Germany	Caucasian	SAH	252/347	36.1/NA	50.4/NA	117/113/22	161/150/36	.903	8
Szpecht 2017	Poland	Caucasian	IH	46/54	58.1/52.2	NA	14/25/7	24/25/5	.677	7
rs1801133 C677T							CC/CT/TT			
Abidi 2018	Morocco	Mixed	HS	113/323	NA	NA	57/45/11	149/139/35	.762	7
Adén 2013	USA	Mixed	IH	224/389	57.1/59.4	NA	98/101/25	165/173/51	.595	7
Aronis 2002	Greece	Caucasian	IH	13/38	47.1/57.9	NA	10/2/1	16/13/9	.072	7
Atadzhanov 2013	Zambia	African	HS	41/116	NA	NA	32/9/0	96/20/0	.301	7
Cai 2005	China	Asian	CH	77/65	57.1/50.8	65.7/62.3	35/24/18	35/19/11	.009	7
Dai 2011	China	Asian	SAH	76/77	31.6/39.0	52.0/54.0	39/36/1	51/20/6	.064	7
Dikmen 2006	Turkey	Caucasian	HS	49/55	54.2/29.1	63.4/56.8	28/18/3	32/21/2	.519	7
Fang 2004	China	Asian	CH	27/96	63.0/59.3	59.9/57.4	10/10/7	40/37/19	.062	7
Fang 2005	Japan	Asian	CH	20/24	57.1/54.2	64.1/64.5	5/12/3	16/6/2	.236	7
Fu 2005	China	Asian	HS	26/79	46.2/48.3	68.6/66.4	10/13/3	37/38/4	.142	7
Hu 2007	China	Asian	CH	32/115	56.3/45.2	57.9/55.3	11/12/9	61/42/12	.249	7
Hu 2016	China	Asian	CH	180/180	70.0/66.7	57.7/57.0	71/81/28	64/86/30	.903	8
Hultdin 2011	Sweden	Caucasian	HS	59/766	78.3/58.6	54.8/55.0	21/30/8	401/306/59	.953	8
Jiang 2018	China	Asian	CH	124/149	60.5/52.3	63.9/62.6	59/50/15	87/51/11	.362	7
Kumar 2018	India	Mixed	SAH	100/50	47.0/52.0	50.5/48.0	32/53/15	39/10/1	.708	7
Li 2003	China	Asian	HS	503/1832	63.3/57.4	58.2/59.6	145/245/113	610/824/398	<.001	8
Nakata 1998	Japan	Asian	CH	35/184	NA	NA	12/18/5	65/83/36	.310	7
Ou 2014	China	Asian	HS	179/1380	59.8/59.9	58.6/60.8	NA	NA	NA	7
Ruigrok 2010	Netherlands	Caucasian	SAH	207/763	28.4/NA	59.5/NA	NA	NA	NA	7
Sazci 2006	Turkey	Caucasian	HS	120/259	55.8/55.6	53.5/56.7	52/52/16	115/119/25	.468	7
Semmler 2008	Germany	Caucasian	SAH	252/346	36.1/NA	50.4/NA	116/109/27	158/152/36	.950	8
Shao 2016	China	Asian	HS	96/206	62.5/64.6	66.2/64.0	33/45/18	64/100/37	.850	7
Somarajan 2011	India	Mixed	HS	215/188	69.8/73.4	57.0/54.0	162/44/9	129/54/5	.818	7
Szpecht 2017	Poland	Caucasian	IH	46/54	58.1/52.2	NA	24/21/1	30/17/7	.090	7
Xiao 2006	China	Asian	CH	61/100	58.0/54.3	55.0/53.0	16/33/12	49/41/10	.742	7
Ye 2004	China	Asian	HS	100/300	NA	NA	63/27/10	192/87/21	.015	7
Zhang 2004	China	Asian	CH	156/239	64.1/38.5	59.2/57.5	51/68/37	65/123/51	.612	7
Zhang 2004	China	Asian	CH	94/100	62.8/51.0	69.7/57.7	21/59/14	40/49/11	.484	7
Zhang 2008	China	Asian	CH	222/282	63.5/37.6	NA	57/103/62	74/140/68	.911	7
Zhao 2001	China	Asian	HS	202/190	NA	NA	88/85/29	55/87/48	.253	7
Zheng 2000	China	Asian	CH	30/122	60.0/52.5	62.0/52.0	17/10/3	62/45/15	.140	7

Abbreviations: CH, cerebral hemorrhage; HS, hemorrhagic stroke; HWE, Hardy–Weinberg equilibrium; IH, intraventricular hemorrhage; NA, not available; NOS, Newcastle–Ottawa scale; SAH, subarachnoid hemorrhage.

### 3.2 MTHFR rs1801131 (A1298C) polymorphism and the risk of ICH

Eight literatures (963 cases and 2,244 controls) assessed relationship between *MTHFR* rs1801131 (A1298C) polymorphism and the risk of ICH. The integrated analyses demonstrated that AA genotype of rs1801131 (A1298C) polymorphism was significantly associated with a decreased risk of IH compared with AC/CC genotypes (OR = 0.63, 95%CI 0.46–0.85; *p* = .003), while AC genotype of rs1801131 polymorphism was significantly associated with an increased risk of IH compared with AA/CC genotypes (OR = 1.55, 95%CI 1.14–2.11; *p* = .005). Additionally, we also found that A allele of rs1801131 polymorphism was significantly associated with a decreased risk of IH compared with C allele (OR = 0.75, 95%CI 0.59–0.95; *p* = .02) (see Table 2).

**Table 2:** Integrated analyses for *MTHFR* gene polymorphisms and intracranial hemorrhage

Population	Sample size Cases/Controls	Dominant comparison (MM vs. Mm + mm)			Recessive comparison (mm vs. MM + Mm)			Overdominant comparison (Mm vs. MM + mm)			Allele comparison (M vs. m)		
		*p* value	OR (95%CI)	*I* ^2^ statistic (%)	*p* value	OR (95%CI)	*I* ^2^ statistic (%)	*p* value	OR (95%CI)	*I* ^2^ statistic (%)	*p* value	OR (95%CI)	*I* ^2^ statistic (%)
rs1801131 A/C													
Overall	963/2244	.07	0.86 (0.73–1.01)	16	.79	1.07 (0.65–1.75)	59	.08	1.16 (0.98–1.36)	16	.16	0.92 (0.81–1.04)	47
Caucasian	526/1482	.58	0.94 (0.76–1.17)	9	.57	1.27 (0.56–2.91)	72	.96	1.01 (0.81–1.25)	0	.59	0.92 (0.69–1.23)	62
HS	341/1404	.82	0.97 (0.75–1.25)	0	.96	1.03 (0.26–3.64)	79	.90	0.98 (0.76–1.27)	0	.91	0.98 (0.69–1.39)	66
SAH	352/397	.86	0.97 (0.72–1.31)	0	.44	0.84 (0.53–1.31)	0	.54	1.10 (0.81–1.48)	0	.76	1.04 (0.83–1.29)	0
IH	270/443	**.003**	**0.63 (0.46–0.85)**	0	.74	1.10 (0.64–1.87)	0	**.005**	**1.55 (1.14–2.11)**	0	**.02**	**0.75 (0.59–0.95)**	0
rs1801133 C/T													
Overall	3679/9067	.05	0.84 (0.71–1.00)	66	.16	1.09 (0.97–1.23)	29	.16	1.07 (0.97–1.16)	44	.13	0.91 (0.80–1.03)	68
Caucasian	746/2281	.43	0.92 (0.74–1.14)	49	.40	1.13 (0.85–1.51)	21	.65	1.05 (0.85–1.30)	21	.73	0.95 (0.72–1.26)	57
Asian	2240/5720	.08	0.83 (0.67–1.02)	62	.27	1.08 (0.94–1.25)	34	.17	1.08 (0.97–1.21)	32	.22	0.91 (0.79–1.06)	67
HS	1703/5694	.95	1.01 (0.81–1.25)	56	.74	1.03 (0.87–1.23)	36	.86	1.01 (0.89–1.15)	4	.78	1.03 (0.86–1.22)	68
SAH	635/1236	.15	0.44 (0.14–1.33)	91	.72	1.14 (0.56–2.30)	58	.12	2.13 (0.81–5.58)	88	.19	0.56 (0.23–1.34)	90
IH	283/481	.50	1.11 (0.82–1.49)	52	.11	0.68 (0.42–1.09)	38	.69	1.06 (0.79–1.43)	43	.20	1.16 (0.93–1.45)	54
CH	1058/1656	**.04**	**0.75 (0.56–0.99)**	59	**.02**	**1.27 (1.03–1.56)**	0	.55	1.05 (0.89–1.23)	32	**.007**	**0.85 (0.76–0.96)**	49

Note

The values in bold represent there are statistically significant differences between cases and controls. All investigated polymorphisms have a major allele (M) and a minor allele (m), the dominant comparison was defined as MM versus Mm + mm, the recessive comparison was defined as mm versus MM + Mm, the overdominant comparison was defined as Mm versus MM + mm, and the allele comparison was defined as M versus m.

Abbreviations: CH, cerebral hemorrhage; CI, confidence interval; HS, hemorrhagic stroke; IH, intraventricular hemorrhage; OR, odds ratio; SAH, subarachnoid hemorrhage.

### 3.3 MTHFR rs1801133 (C677T) polymorphism and the risk of ICH

Thirty‐one literatures (3,679 cases and 9,067 controls) assessed relationship between *MTHFR* rs1801133 (C677T) polymorphism and the risk of ICH. The integrated analyses demonstrated that CC genotype of rs1801133 (C677T) polymorphism was significantly associated with a decreased risk of CH compared with CT/TT genotypes (OR = 0.75, 95%CI 0.56–0.99; *p* = .04), while TT genotype of rs1801133 polymorphism was significantly associated with an increased risk of CH compared with CC/CT genotypes (OR = 1.27, 95%CI 1.03–1.56; *p* = .02). Additionally, we also found that C allele of rs1801133 polymorphism was significantly associated with a decreased risk of CH compared with T allele (OR = 0.85, 95%CI 0.76–0.96; *p* = .007) (see Table 2).

### 3.4 Sensitivity analyses

We tested stability of quantitative analysis results by deleting one eligible study each time, and then recalculating the pooled results based on the rest of studies. The overall trends of associations remained unchanged in sensitivity analyses, which indicated that our quantitative analysis results were quite reliable and stable (Relevant datasets can be found at https://osf.io, username: j7ffofnjwi@163.com , password: j7ffofnjwi @).

### 3.5 Publication biases

We estimated potential publication biases in this meta‐analysis with funnel plots and Egger's tests. Funnel plots were found to be overall symmetrical (Figure S1). We further calculated *p* values for Egger's tests and found that *p* values were >.05 in all comparisons (for rs1801131 polymorphism: dominant comparison, *p* = .429; recessive comparison, *p* = .277; overdominant comparison, *p* = .520; allele comparison, *p* = .481; for rs1801133 polymorphism: dominant comparison, *p* = .164; recessive comparison, *p* = .372; overdominant comparison, *p* = .763; allele comparison, *p* = .325). These results indicated that our pooled quantitative analysis results were not likely to be seriously influenced by publication biases.

### 3.6 Meta‐regression analysis

Meta‐regression analysis was performed to determine whether study quality of eligible studies can change the effect size, and the findings of meta‐regression analysis indicated that the effect sizes of pooled analyses were not altered by study quality (see Table 3).

**Table 3:** Meta‐regression analysis for study quality

Heterogeneity factor (Study quality)	Coefficient	*SE*	*T* test	*p* value	95% CI	
					UL	LL
rs1801131 A1298C						
Dominant comparison	0.067	0.035	1.84	.11	−0.011	0.156
Recessive comparison	0.021	0.033	0.67	.63	−0.047	0.093
Overdominant comparison	0.039	0.024	1.49	.18	−0.016	0.112
Allele comparison	0.061	0.049	1.33	.22	−0.049	0.141
rs1801133 C677T						
Dominant comparison	0.089	0.041	1.71	.09	−0.015	0.199
Recessive comparison	0.023	0.015	1.28	.27	−0.022	0.067
Overdominant comparison	0.051	0.028	1.64	.13	−0.014	0.125
Allele comparison	0.042	0.037	0.96	.34	−0.037	0.132

Abbreviations: LL, lower limit; *SE*, standard error; UL, upper limit.

## 4 DISCUSSION

This meta‐analysis, robustly estimated associations between gene polymorphisms in *MTHFR* and the risk of ICH. It is so far the very first meta‐analysis regarding rs1801131 (A1298C) polymorphism and ICH, and it is also so far the most complete meta‐analysis regarding rs1801133 (C677T) polymorphism and ICH. The pooled analyses results showed that rs1801131 (A1298C) polymorphism was significantly associated with the risk of IH, whereas rs1801133 (C677T) polymorphism was significantly associated with the risk of CH.

The following points need be taken into consideration when interpreting our pooled analyses. First, previous experimental studies have found that those two investigated *MTHFR* polymorphisms may alter enzymatic function of MTHFR and raise plasma homocysteine levels, which may attribute to a higher risk of ICH (Li et al., 2015; Santilli et al., 2016; Trimmer, 2013; Zaric et al., 2019). Nevertheless, further experimental studies still need to explore the exact molecular mechanisms of the observed positive associations between *MTHFR* gene polymorphisms and the risk of certain types of ICH in the current meta‐analysis. Second, we aimed to study all polymorphic loci of *MTHFR* gene. Nevertheless, due to paucity of relevant publications, only those two most commonly investigated polymorphisms of *MTHFR* gene can be pooled analyzed in this meta‐analysis. Third, it is notable that previously, Gao et al. (2012) and Hu et al. (2016) also tried to investigate associations between *MTHFR* rs1801133 (C677T) polymorphism and ICH through a meta‐analysis. Nevertheless, the previous meta‐analysis by Gao et al only covered relevant genetic association studies that were published before 2011, while the previous meta‐analysis by Hu et al only focused on studies that were conducted in the Chinese population, and two recent publications in the Chinese population were not covered by Hu and colleagues (Jiang, Sheng, & Luo, 2018; Shao, Meng, & Wu, 2016). In contrast to those two previous meta‐analyses, we did not observe positive associations with ICH in overall population and Asians. Positive results were only detected for rs1801133 (C677T) polymorphism in the CH subgroup in our integrated analyses, but not in other types of ICH. Considering that our integrated analyses were derived from more eligible studies, our observations should be more statistically robust. Nevertheless, further studies with larger sample sizes are still warranted to confirm our findings. Actually, if we set type I error at 5%, set the power at 80%, and set the estimated between group difference at 10%, a sample size of around 950 would be sufficient to detect the differences between two groups. In the current meta‐analysis, only the pooled sample sizes of SAH and IH subgroups for rs1801131 as well as IH subgroup for rs1801133 did not reach these criteria, so the findings observed in those subgroups should be considered as exploratory and future studies still need to test the robustness of those pooled results.

Despite being the so far most comprehensive meta‐analysis on this topic, it should be acknowledged that the present study also has two major limitations. Firstly, our quantitative analysis results were derived from unadjusted pooling of previous publications due to lack of access to raw data of eligible studies. Without genotypic data according to age, sex or comorbid status, it is impossible for us to conduct adjusted analyses accordingly. Although almost all meta‐analyses shared the same limitation, it should be acknowledged that lack of further adjustment for baseline characteristics such as age, sex or comorbid status may somehow influence reliability of our findings (Chen, Chang, & Chen, 2018; Falcone & Woo, 2017). Secondly, environmental factors such as diets or exercise levels may also influence associations between *MTHFR* polymorphisms and the risk of ICH. However, most of previous publications failed to consider environmental factors, so it is impossible for us to explore genetic–environmental interactions in a meta‐analysis based on previous publications (Ma et al., 2015).

In conclusion, this meta‐analysis shows that rs1801131 (A1298C) polymorphism may affect the risk of IH, whereas rs1801133 (C677T) polymorphism may affect the risk of CH. Nevertheless, further studies with larger sample sizes are still needed to confirm our findings.

## CONFLICT OF INTEREST

None declared.

## AUTHORS' CONTRIBUTIONS

Fenghui Wang and Youbin Jing conceived and designed this meta‐analysis. Fenghui Wang and Zhendong Xu searched literatures. Haiyan Jiao and Aixiang Wang analyzed data. Fenghui Wang and Youbin Jing wrote the manuscript. All authors have approved the final manuscript as submitted.

### Peer Review

The peer review history for this article is available at https://publons.com/publon/10.1002/brb3.1840.

## Supporting information

FigS1

## Data Availability

Data sharing not applicable to this article as no datasets were generated or analyzed during the current study.
