# Supplementary material for: Associations between MTHFR gene polymorphisms and the risk of intracranial hemorrhage: Evidence from a meta‐analysis
Source: Brain Behav. 2020 Nov 27;11(1):e01840. doi: 10.1002/brb3.1840 (PMC7821613; doi:10.1002/brb3.1840)
Supplement: Supplementary file 1 — FigS1 [file BRB3-11-e01840-s001.docx]

**Supplementary Figure 1. Funnel plots of investigated polymorphisms**


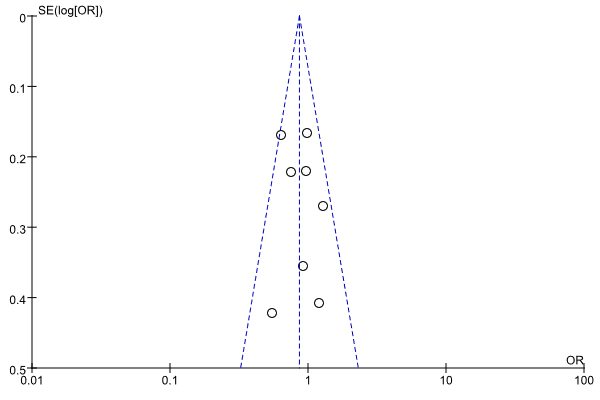


**Funnel plot of rs1801131 polymorphism and ICH under dominant comparison**


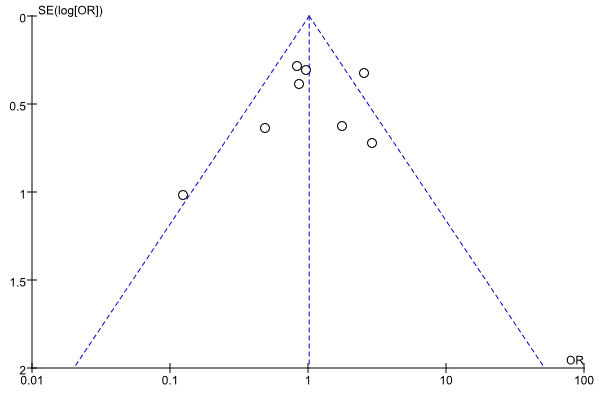


**Funnel plot of rs1801131 polymorphism and ICH under recessive comparison**


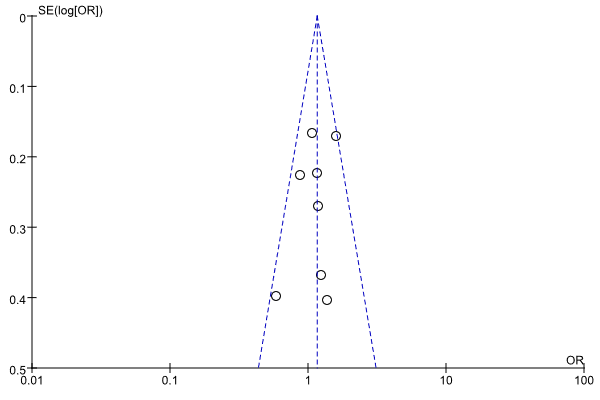


**Funnel plot of rs1801131 polymorphism and ICH under over-dominant comparison**


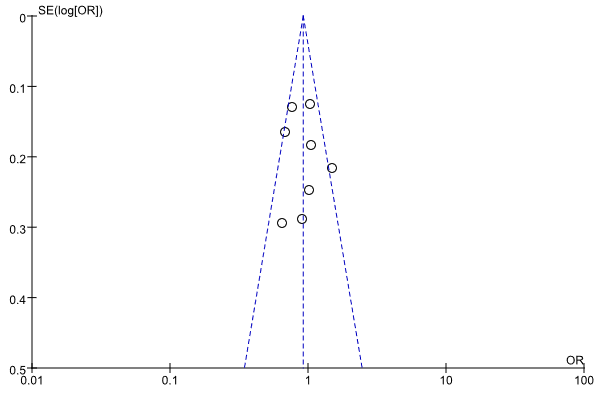


**Funnel plot of rs1801131 polymorphism and ICH under allele comparison**


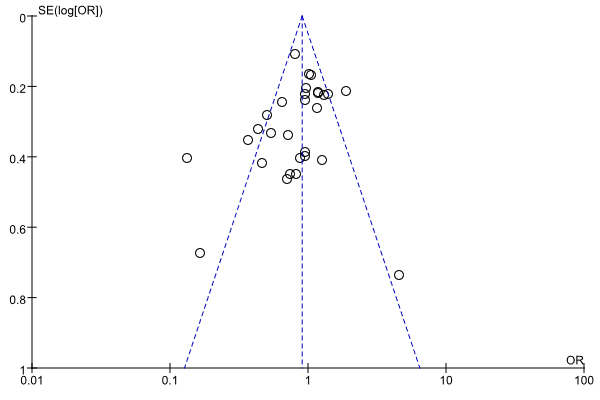


**Funnel plot of rs1801133 polymorphism and ICH under dominant comparison**


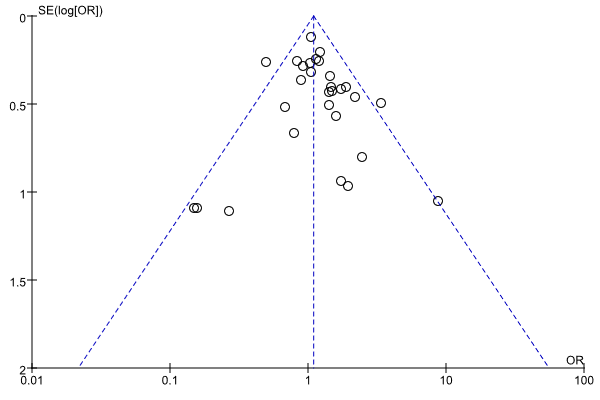


**Funnel plot of rs1801133 polymorphism and ICH under recessive comparison**


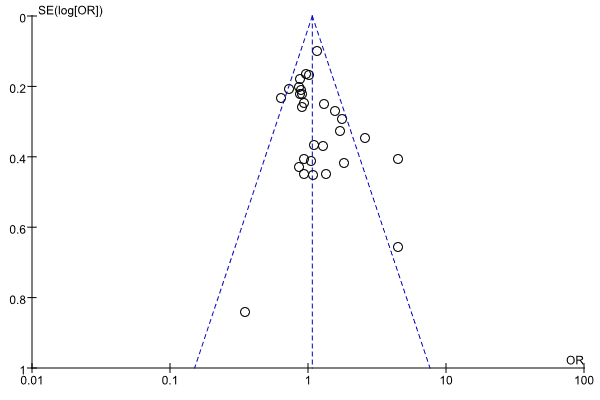


**Funnel plot of rs1801133 polymorphism and ICH under over-dominant comparison**


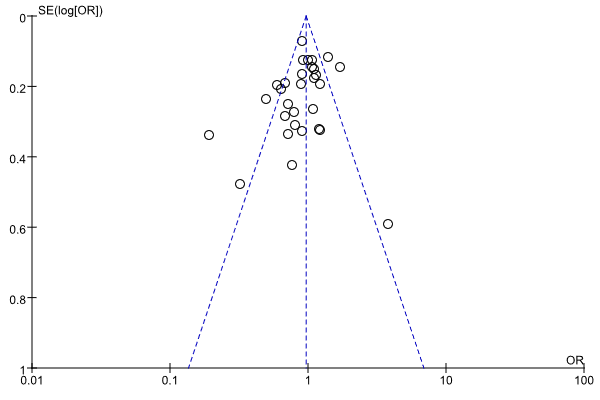


**Funnel plot of rs1801133 polymorphism and ICH under allele comparison**
